# Oncogenic driver mutations, treatment, and EGFR-TKI resistance in a Caucasian population with non-small cell lung cancer: survival in clinical practice

**DOI:** 10.18632/oncotarget.20857

**Published:** 2017-09-13

**Authors:** Martin Faehling, Birgit Schwenk, Sebastian Kramberg, Robert Eckert, Anna-Lena Volckmar, Albrecht Stenzinger, Jörn Sträter

**Affiliations:** ^1^ Department of Cardiology and Pneumology, Hospital Esslingen, Esslingen, Germany; ^2^ Outpatient Cancer Treatment Clinic Esslingen, Esslingen, Germany; ^3^ Institute of Pathology, University Hospital Heidelberg, Heidelberg, Germany; ^4^ Institute of Pathology Esslingen, Esslingen, Germany

**Keywords:** NSCLC, EGFR, ALK, BRAF, overall survival

## Abstract

**Introduction:**

Oncogenic driver mutations activating *EGFR*, *ALK*, or *BRAF* in NSCLC predict sensitivity to specific tyrosine-kinase inhibitors (TKIs). We provide data on prevalence, treatment and survival of driver-mutation positive NSCLC in a predominantly Caucasian population in routine clinical practice.

**Patients and Methods:**

NSCLC patients diagnosed from 2006-2015 with an EGFR-test result were included (n=265). Testing for *EGFR*, *ALK*, or *BRAF* was performed if specific TKI therapy was considered. Case-control analyses of overall survival (OS) comparing driver-mutation positive and negative patients were performed.

**Results:**

44 sensitizing *EGFR* mutations (17%), 8 *ALK* translocations (7%, n=111) and 3 *BRAF* mutations (8%, n=39) were detected in adenocarcinoma or adenosquamous carcinoma. We did not find mutations in tumors without an adenocarcinoma-component. More than 90% of inoperable driver-mutation positive patients received TKI-therapy. Case-control analysis revealed improved OS of driver-mutation positive patients (39.6 vs. 19.4 months, HR 0.51). OS was improved in stage IV patients but not in stage I-III patients.

OS of EGFR-TKI treated patients was similar for 1^st^ and 2^nd^-line EGFR-TKI treatment. Patients not treated with EGFR-TKI had no benefit in OS. Re-biopsies obtained at progression revealed an *EGFR*-T790M mutation in 73% (n=11). These patients responded to the 3^rd^-generation EGFR-TKI osimertinib.

**Discussion:**

Testing guided by predictive clinical parameters resulted in twice as high rates of mutation-positive patients than expected, and TKI treatment resulted in a strong long-term OS advantage.

**Conclusion:**

Testing for driver mutations is feasible in routine clinical practice, and identifies patients who benefit from TKI-therapy. OS compares favorably with OS in clinical studies.

## INTRODUCTION

Lung cancer is the main cause of cancer-related mortality in men and second to breast cancer in women. The majority of patients is diagnosed at an advanced stage with 5-year survival rates with conventional chemotherapy regimens of about 5%. The most common type is non-small cell lung cancer (NSCLC) with adenocarcinoma (AC) as the main subtype with rising incidence. A subgroup of NSCLC harbors an oncogenic driver, in particular activating *EGFR* mutations, *ALK*- or *ROS1* translocations, or *BRAF* mutations. These oncogenic drivers are almost exclusively present in AC. In 2004, it was reported that activating mutations in *EGFR* predict response to specific EGFR-tyrosine kinase inhibitors (TKIs) in NSCLC patients [1, 2]. In the following, these EGFR-TKI sensitizing mutations are referred to as “*EGFR*-positive”.

Unlike conventional chemotherapy reagents, TKI are mainly cytostatic compounds that interfere with a specific mechanism driving tumor growth. Several trials confirmed the superiority of the reversible 1^st^ generation EGFR-TKIs gefitinib and erlotinib, and the irreversible 2^nd^ generation EGFR-TKI afatinib over cytotoxic chemotherapy in terms of response and progression-free survival (PFS) both in pretreated and untreated *EGFR*-positive NSCLC [3, 4, 5, 6]. In *ALK*-rearranged NSCLC, the ALK-TKIs crizotinib, ceritinib, and more recently alectinib have shown superior response and PFS compared to chemotherapy irrespective of treatment line [7, 8, 9, 10]. Furthermore, uncontrolled studies showed high response rates to crizotinib and ceritinib in *ROS1*-rearranged NSCLC [11, 12], and to dabrafenib and vemurafenib (more recently combined with a MEK-inhibitor, e. g. trametinib) in *BRAF*-mutated (*V600E*) NSCLC [13, 14, 15]. Thus, the presence of a driver mutation predicts response to specific TKIs. Moreover, EGFR-positive NSCLC and probably *ROS1*-rearranged NSCLC are associated with improved prognosis [16, 17], whereas this is appears not to be the case for *ALK*- or *BRAF*-positive NSCLC [18, 19].

Mainly due to crossover, a benefit of EGFR-TKI therapy in *EGFR*-positive NSCLC on overall survival (OS) is hard to demonstrate in the context of prospective randomized trials [20, 21, 22, 23]. Thus, prospective clinical trials are of limited value for the assessment of the effect of TKI-therapy on OS [24]. However, retrospective analyses of OS of patients with advanced inoperable EGFR-positive NSCLC on EGFR-TKI-therapy reported a median OS of 25-31 months and a 5-year survival of 15-20% [25, 26, 27]. These reports together with historic comparisons of survival of EGFR-mutation positive patients before and after availability of EGFR-TKI therapy [28, 29], post-hoc pooled survival analysis of patients with EGFR-TKI sensitive NSCLC (del exon 19 only) [20], and prospective non-randomized data from the Lung Cancer Mutation Consortium [30] have strengthened the general assumption that EGFR-TKI-therapy also improves OS. However, a controversy remains as to whether 1^st^ line EGFR-TKI therapy confers an OS advantage compared to 2^nd^ line EGFR-TKI therapy [31]. A survival advantage was reported for 1^st^ line EGFR-TKI therapy in a Chinese retrospective analysis [32]. This contrasts with findings from the nationwide French survival data recently presented pointing to an OS benefit of EGFR-mutation positive patients treated with EGFR-TKI 2^nd^ line [33] and with recent clinical evidence of poorer efficacy of chemotherapy after EGFR-TKI [34]. The only study directly comparing a second generation EGFR-TKI (afatinib) with a first generation EGFR-TKI (gefitinib) as first-line therapy did not reveal a difference in OS [35]. Despite good response to TKI therapy in driver-mutation positive NSCLC, resistance develops after generally less than one year. In the case of *EGFR*-positive NSCLC, resistance is due to the de novo mutation T790M in *EGFR* exon 20 in more than 50% of cases [36]. The 3^rd^ generation EGFR-TKI osimertinib has recently been shown to be active in T790M-mutated NSCLC [37].

Most studies on driver-mutation positive NSCLC are based on Eastern Asian populations because of a higher incidence [38]. ‘Real world data’ from routine clinical practice on driver mutations, treatment, and long-term survival in Caucasian lung cancer patients are still scarce. Such data are, however, important to assess performance of precision medicine approaches in daily clinical practice and to guide testing as well as clinical management and therapy of NSCLC patients. To bridge this gap, we here report prevalence and distribution of driver mutations, treatment modalities, and analysis of resistance mechanisms in a current predominantly Caucasian patient population and performed a retrospective case-control analysis of OS.

## RESULTS

### Baseline characteristics

Eight-hundred twenty-four patients were diagnosed with NSCLC from 2006-2015. A detailed analysis of the full patient cohort is in preparation and will be reported elsewhere. An *EGFR*-mutation test result was available for 322 patients (39%). In none of the 57 non-AC patients with *EGFR*-test results, an activating *EGFR* mutation was detected. The following analysis focuses on 265 patients with AC histology or adenosquamous histology for whom *EGFR*-test results were available (59.8% of all AC patients). Of these patients, 188 (70.9%) had died at databank lock. Of the 77 living patients, 71 (92.2%) had attended the last scheduled follow-up examination (at least once a year until five years after diagnosis). Thus, follow-up was complete for 259 patients (97.7%). Median follow-up of living patients was 36.6 months.

Baseline clinical characteristics and results of molecular testing are given in Table 1. The *EGFR*-tested population had a better performance status and included a higher proportion of women (51% of tested patients, 46% of all AC patients) and of never-smokers (29% of tested patients, 21% of all AC patients) than the unselected AC population. 84% of never-smokers had an *EGFR*-test result compared to 60% of the unselected AC population. Histological grading of the tumor influenced the likelihood of being tested for *EGFR*-mutational status (Table 2). Whereas 76% of well differentiated G1 tumors had a test result available, this was the case for only 55-59 % of less well differentiated tumors or tumors for which no grading was reported.

**Table 1:** Clinical characteristics of unselected AC patients, AC patients with driver-mutation test result, and case-control patients for analysis of driver-mutation status and survival

Oncogenic driver mutation	Adenocarcinoma (AC)		Unselected population with EGFR-test result		Case-control patients	
	All patients	EGFR-tested patients	Driver-mutation positive	Driver-mutation negative	Driver-mutation positive	Driver-mutation negative
**n**	443	265	55	210	49	
**Age (mean, range)**	67.3 (38.8-94.7)	67.1 (38.8-88.2)	68.7 (38.8-87.0)	66.7 (44.6-88.2)	68.2 (38.8-84.3)	67.6 (45.0-86.2)
**Deceased**	316 (71%)	188 (71%)	35 (64%)	153 (73%)	30 (64%)	34 (72%)
***Gender***						
Male	240 (54%)	129 (49%)	18 (33%)	111 (53%)	16 (33%)	
Female	203 (46%)	136 (51%)	37 (67%)	99 (47%)	33 (67%)	
***Stage (UICC 7^th^ ed.)***						
IA/B	49/41 (20%)	17/18 (13%)	3/5 (15%)	13/13 (13%)	3/4 (14%)	
IIA/B	22/19 (9%)	12/13 (9%)	2/3 (9%)	10/9 (9%)	1/3 (8%)	
IIIA/B	41/28 (16%)	23/16 (15%)	5/3 (15%)	18/13 (15%)	4/3 (14%)	
IV M1a/M1b	87/156 (55%)	61/105 (63%)	11/23 (62%)	49/82 (63%)	12/19 (63%)	
***Performance status***	n. a. 1					
ECOG 0	128 (29%)	90 (34%)	24 (44%)	66 (31%)	21 (43%)	
ECOG 1	219 (50%)	133 (50%)	24 (44%)	109 (52%)	24 (49%)	
ECOG 2	72 (16%)	36 (14%)	6 (11%)	30 (14%)	4 (8%)	
ECOG 3	19 (4%)	6 (2%)	1 (2%)	5 (2%)	0	
ECOG 4	4 (1%)	0	0	0	0	
***Smoking status***	n. a. 16	n. a. 2		n. a. 2		
Never-smoker	91 (21%)	76 (29%)	39 (71%)	37 (18%)	34 (69%)	
Long-term ex-smoker	156 (37%)	101 (39%)	10 (18%)	91 (44%)	10 (20%)	
Quitter	92 (22%)	58 (22%)	6 (11%)	52 (25%)	5 (10%)	
Smoker	88 (21%)	28 (11%)	0	28 (13%)	0	
***EGFR mutation***						
EGFR status known	265	265	55	210	49	
EGFR positive	44 (17%)	44 (17%)	44	0	39 (80%)^a^	0
*Del Exon19*	*27^b^*	*27^c^*	*27*	*-*	*25*	*-*
*Exon21 L858R*	*12*	*12*	*12*	*-*	*9*	*-*
*Exon18*	*5*	*5*	*5*	*-*	*5*	*-*
***ALK translocation***						
ALK status known	111	111	17	94	13	22
ALK positive	8 (7%)	8 (7%)	8	0	7 (14%)^b^	0
***BRAF***						
BRAF status known	39	39	6	33	5	7
BRAF pos. (V600E)^d^	3 (8%)	3 (8%)	3	0	3 (6%)^b^	0

n. a.: not assessed.

a Percentage of driver-positive case-control population.

b Including one patient with dupExon19 mutation.

c Including one patient with dupExon19 mutation.

d excluding one EGFR-mutation positive patient with BRAF mutation detected after EGFR-TKI treatment as a resistance mutation.

Patients were matched for gender, performance status, clinical stage, smoking status, and age. All patients for oncogenic-driver analysis had AC histology since no driver mutations were detected in other histologies. Matched parameters are given as a single column.

**Table 2:** Histopathological grading, EGFR-testing and EGFR-positivity in patients with AC

Grade	All AC patients (n=443)	EGFR tested (n=265)	EGFR-positive (n=44)
**G1**	38 (8.6%)	29 (10.9%)	9 (20.5%)
**G2**	97 (21.9%)	55 (20.8%)	15 (34.1%)
**G3**	248 (56.0%)	146 (55.1%)	12 (27.3%)
**No grade given**	50 (11.3%)	28 (10.6%)	4 (9.1 %)
**Adenosquamous**	10 (2.3%)	7 (2.6%)	4 (9.1%)

### Oncogenic driver mutations: distribution

**Figure 1:**
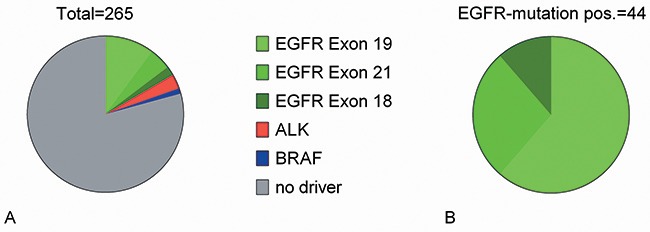
Oncogenic-driver mutation: distribution **(A)** Distribution of oncogenic driver mutations in AC patients with EGFR-test result. **(B)** Distribution of *EGFR* mutations.

Driver mutations were detected in 55 patients of 265 tested AC patients (20.8%). The distribution and frequency of the 55 patients with an oncogenic driver mutation are shown in Figure 1A. Sensitizing *EGFR*-mutations were most prevalent (79%), followed by *ALK*-translocations (15%) and the *BRAF* mutation V600E (6%).

*EGFR:* Forty-eight *EGFR* mutations were detected. Forty-four were mutations known to be sensitizing [39, 40, 41, 42]. Four mutations were rare point mutations:

· Transition exon 19 c.2203G>A; p.G735S in a female ex-smoker with AC G3 stage IV (M1b). This mutation has been described twice in lung cancer (COSMIC databank accessed 31.10.2016) with no data on response to EGFR-TKI therapy given in the literature [43, 44]. The patient showed progressive disease on 2^nd^ line EGFR-TKI therapy with gefitinib.

· Transition exon 19 c.2258T>C; p.P753L in a female smoker with SCC stage IIIA. This mutation has not been described previously in lung cancer (COSMIC databank search 31.10.2016). Upon recurrence after resection, the patient was treated 1^st^-line with erlotinib with early progression after 14 weeks of disease stabilization.

· Transition exon 21 c.2543C>T; p.P848L in a male ex-smoker with AC G1 stage IV (M1b). This patient showed stable disease on erlotinib with a relatively short PFS of 4.6 months. This mutation has been previously described in nine lung samples (COSMIC databank, accessed 31.10.16) and was shown to be non-activating [45].

· Transition exon 21 c.2527G>A; p.V843I mutation in an ex-smoker with NSCLC (NOS) who did not receive targeted EGFR-TKI therapy. This mutation has been reported twice in lung cancer (COSMIC databank, accessed 31.10.16) and is activating from a biological point of view but does not confer sensitivity to EGFR-TKIs [46, 47].

Such point mutations are typically smoking-induced [48]. Since these four mutations do not confer EGFR-TKI sensitivity, they were counted as *EGFR*-negative for the survival analysis of *EGFR*-tested patients. Thus, the prevalence of *EGFR*-positive patients was 16.6% in the tested population. The majority had a del-exon 19 mutations followed by exon 21 point mutation and exon 18 mutations (Figure 1B, Table 3). While 22.8% of tested women were *EGFR*-positive, only 10.1% of men had an *EGFR*-positive NSCLC. *EGFR*-positive patients were older than *EGFR*-negative patients (70.2 vs. 66.5 years, p=0.038). 31% of tested G1 tumors and 28% of G2 tumors were *EGFR*-positive, compared to only 8% of G3 tumors (Table 2). In line with a previous report [49], the few patients with adenosquamous carcinoma and an *EGFR*-test result (n=7) had a high likelihood of a positive result (57%). Based on conventional staining, two of the patients with adenosquamous histology had been initially classified as squamous-cell carcinoma (SCC). However, re-evaluation of these cases including TTF1 immunohistochemistry revealed areas with AC differentiation. None of 36 current smokers tested for *EGFR*-mutation was EGFR-positive.

**Table 3:** Patient characteristics and type of EGFR mutation, and EGFR-TKI treatment of EGFR-positive patients

	Exon 19			Exon 21		Exon 18
	del Exon19	dup Exon19	Exon19 point mutations (G735S; P753L), non-activating	L858R	V843I, activating but not sensitizing; P848L, non-activating	G719A: 1G719C: 2E709_T710>D: 1complex: 1(E709A + G719S)
n	26	1	2	12	2	5
age (mean)	70.1	65.7	60.9	70.7	56.8	70.5
female/male	18/8	0/1	2/0	10/2	1/1	3/2
smoking status^1^	0: 18;1: 8	0: 1	1: 12: 1	0: 81:4	1: 2	0: 41: 1
histology	AC 26	AC 1	AC 1, SCC 1	AC 12	AC 1, NOS 1	AC 5
TKI treated	23^2^	1	2	8^3^	1	5
ECOG^4^	0: 91: 102: 4	1: 1	1: 2	0: 21: 52: 1	0: 11: 1	0: 31: 2
Stage(UICC 7^th^ ed.)	I-III: 5^5^IV M1a: 5IV M1b: 13	IV M1b: 1	IV M1a: 1IV M1b: 1	IV M1a: 2IV M1b: 6	IV M1a: 1IV M1b: 1	IV M1a: 3IV M1b: 2
BRA^6^	6	1	0	4	1	0
OSS^7^	13	1	0	4	0	2
other M1b site	4	1	1	0	0	0
line TKI	1^st^: 152^nd^: 8	1^st^:1	2^nd^: 2	1^st^: 62^nd^: 2	1^st^:1	1^st^: 32^nd^: 2
1^st^ TKI^8^	A 1E 19G 3	E 1	E 1G 1	A 1E 7	E 1	A 1E 4
response^9^ to 1st TKI	CR 1PR 21SD 1	SD 1	SD 1PD 1	PR 8	SD 1	PR 4SD 1
progressionon TKI^10^	0: 81: 15	1: 1	1: 2	0: 21: 6	1: 1	0: 11: 4
brain as site of 1st progression	3	1	0	2	0	1
local therapy at progression^11^	RT 5	RT 1	0	RT 2	RT 1	RT 1
1^st^ chemotherapy (carboplatinum-gemcitabine)^12^	7	1	1	4	1	1
response to first chemotherapy^6^	PR 3SD 3PD 1	PD 1	PR 1	PR 2SD 1PD 1	PR 1n. a. 1	PD 1
subsequent chemotherapies^13^	Pem 5Doce 1Gem 1	0	0	Pem 2Pac 1Vin 1	Pem, Doce	Pem 4Gem 1
response to subsequent chemotherapies^6^	PR 2SD 2PD 2, n.a. 1	-	-	PR 1SD 2PD 1	PD 1, SD 1	PR 2SD 3
switch TKI^14^	A 4E 1G 1	A 1	E 1	A 2G 1	-	-
response toswitch TKI^6^	PD 6	PR 1	PD 1	PD 3	-	-
T790M positive at progression^15^	7 (n=8)	n. a.	n. a.	n. a.	n. a.	1 (n=3)
3rd gen TKI	7	-	-	-	-	1

^1^ 0: never smoker, 1: ex-smoker, 2: smoker.

^2^ Performance status at start of TKI.

^3^ BRA: Brain metastases present at start of TKI-therapy.

^4^ OSS: Bone metastases present at start of TKI-therapy.

^5^ A: afatinib; E: erlotinib; G: gefitinib.

^6^ Number of patients with complete remission (CR), partial remission (PR), stable disease (SD), or progressive disease (PD).

^7^ 0: no progression; 1: progression at last observation.

^8^ RT: radiotherapy (number of patients).

^9^ EGFR-TKI holiday during platinum doublett chemotherapy.

^10^ No EGFR-TKI holiday during subsequent mono-chemotherapies. Pem: pemetrexed; Gem: gemcitabine; Doce: docetaxel; Pac: paclitaxel.

^11^ Number of patients receiving another 1^st^/2^nd^ generation EGFR-TKI after progression on TKI.

^12^ Number of patients with positive test result (n: number of patients tested).

^13^ Three localized stage operable patients (IB, IIB, IIIA) did not have a recurrence and did not receive EGFR-TKI therapy.

^14^ Five functionally inoperable localized stage patients (IB (3), IIIA, IIIB) received palliative EGFR-TKI therapy.

^15^ One localized stage operable patient (IIIA) did not have a recurrence and did not receive EGFR-TKI therapy. Four stage IV patients did not receive EGFR-TKI therapy because of poor performance state.

*ALK/ROS1/BRAF:* 111 patients (97% *EGFR* negative) were tested for an ALK translocation with 8 positive results (7%). Five of 56 men (8.9%), and 3 of 54 women (5.6%) had a positive *ALK*-FISH test. In line with the literature [50], patients with an ALK-translocation tended to be younger than the *ALK*-negative patients (60.7 vs. 65.7 years, p=0.17). In keeping with the concept of mutual exclusiveness of classic mutated driver genes, none of the four *EGFR*-positive patients who also underwent *ALK*-testing had an *ALK*-translocated tumor [51]. Among 35 patients (all *EGFR-* and *ALK-*negative) tested for *ROS1* translocation, none was detected. Among 39 patients tested for *BRAF* mutations, the classic V600E mutation was detected in three patients (8%). All of the three *BRAF*-positive patients were women (n=16, 19% positive), whereas none of 22 men tested had a *BRAF* mutation.

### Oncogenic driver mutations: OS by stage and mutation

**Figure 2:**
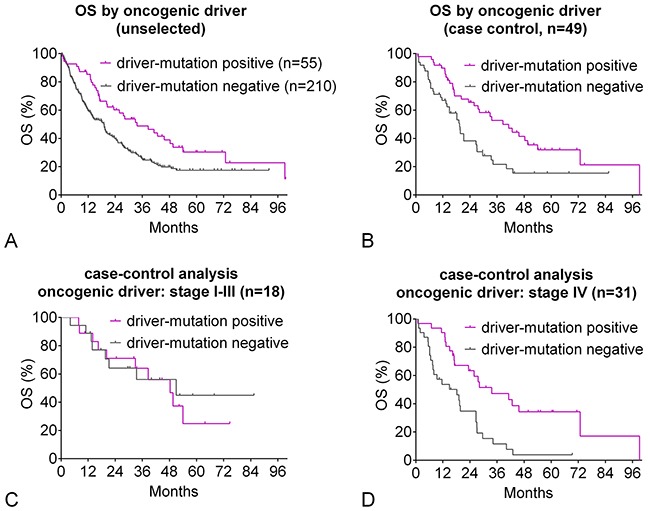
Oncogenic-driver mutation: overall survival **(A)** Unselected patients: Kaplan-Meier curves for OS of driver-mutation positive patients compared to patients with no driver mutation detected. Clinical characteristics are given in Table 1. **(B-D)** Case-control analysis: Patients were matched for gender, clinical stage, performance status, smoking status, and age. Clinical characteristics are given in Table 1. **(B)** Kaplan-Meier curves for OS of driver-mutation positive patients compared to patients with no driver mutation detected. **(C)** Kaplan-Meier curves for OS of driver-mutation positive patients stage I-III compared to patients with no driver mutation detected. **(D)** Kaplan-Meier curves for OS of driver-mutation positive patients stage IV compared to patients with no driver mutation detected.

**Figure 3:**
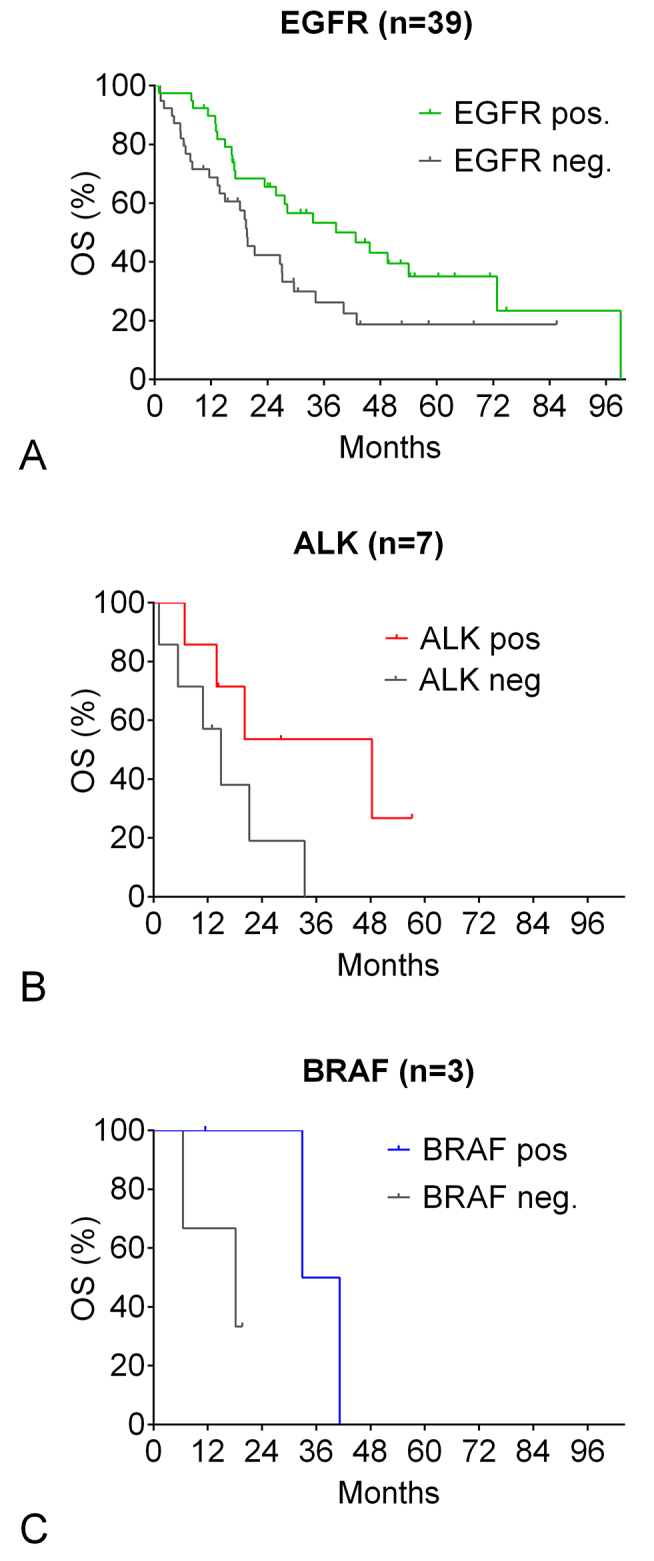
Oncogenic-driver mutation: overall survival by specific mutation **(A-C)** Kaplan-Meier curves for OS of driver-mutation positive patients compared to patients with no driver mutation detected (case-control analysis). Patients were matched for gender, clinical stage, performance status, smoking status, and age. (A) *EGFR*-positive patients. (B) *ALK*-positive patients. (C) *BRAF*-positive patients.

The unselected driver-mutation positive population had a significantly longer OS of 33.6 months compared to 18.9 months in driver-mutation negative patients (Hazard ratio [HR] 0.60, CI 0.45 - 0.86, p=0.0045 [Figure 2A]). The case-control analysis confirmed a significantly superior OS in the driver-mutation positive population of 39.6 months compared to 19.4 months in driver-mutation negative patients (HR 0.51, CI 0.29 - 0.79, p=0.021 [Figure 2B]). Case-control analysis by stage revealed no difference in OS in stage I-III patients but a highly significant benefit in OS in the stage IV driver-positive population (HR 0.39, CI 0.18 - 0.61, p=0.0015 [Figure 2C, 2D]). In this population, 5-year survival of driver-positive patients was 34.3% compared to 3.9% of driver-negative patients. Median OS of the case-control subpopulations compared to the corresponding driver-negative controls was 39 vs. 20 months for *EGFR*-positive patients (HR 0.55, CI 0.30 – 0.92, p=0.016, n=37), 48 vs. 15 months for *ALK*-positive patients (HR 0.39, CI 0.09 - 1.21, p=0.11, n=7), and 37 vs. 18 months for *BRAF*-positive patients (HR 0.33, CI 0.014 – 1.30, p=0.166, n=3) (Figure 3A-3C).

### Oncogenic driver mutations: treatment

**Figure 4:**
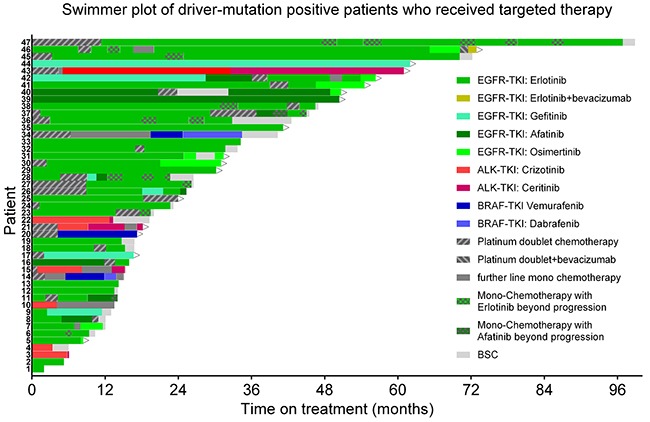
Oncogenic driver mutation-positive patients: treatment course Swimmer plot showing the sequence of treatment lines in patients who received at least one line of targeted therapy. Bars start from begin of palliative treatment. Arrows signify ongoing therapy. Green: EGFR TKIs. Red: ALK TKIs. Blue: BRAF TKIs. Dark gray: Chemotherapy.

Forty-seven of 55 patients with a sensitizing driver mutation received targeted therapy. Their treatment course is shown in the swimmer plot (Figure 4). Eight driver-positive patients did not receive targeted therapy either because they had localized disease and received curative treatment without recurrence (n=4) or because of poor performance state (n=4). The swimmer plot shows that in line with the outlined treatment principles, most patients received both targeted therapy and chemotherapy. The patient surviving longest (99.2 months) was a male patient with stage IV disease at diagnosis and a complex EGFR exon18 mutation (E709A and G719S). Because of the small numbers of *ALK*- and *BRAF*-positive patients, the survival analysis focuses on *EGFR*-positive patients.

Thirty-seven of the 44 *EGFR*-positive patients received EGFR-TKI therapy (Figure 4, Table 3). PFS of unselected *EGFR*-positive patients on first or second generation EGFR-TKI (gefitinib, erlotinib, or afatinib) was 16.9 months with no difference between 1^st^ line TKI therapy (16.9 months) and 2^nd^ line therapy (18.5 months). The total time on TKI therapy was longer (26.2 months), reflecting EGFR-TKI therapy beyond progression. OS of TKI-treated *EGFR*-positive patients was 38.6 months.

### EGFR-mutation: OS by subpopulation

**Figure 5:**
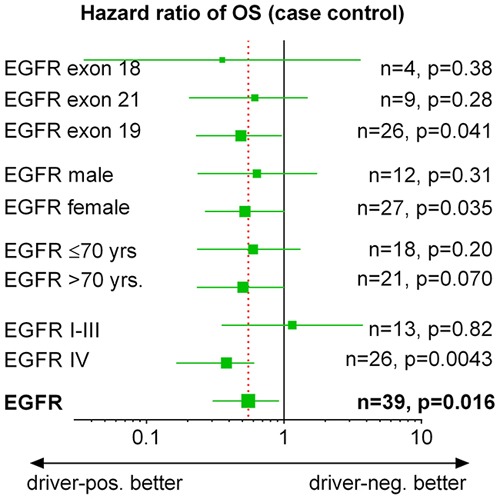
*EGFR*-positive patients: subgroup analysis of overall survival Forrest plot showing HR and CI of subgroups of the case-control population in Figure 3A. I-III: UICC stage I-III, IV: UICC stage IV. p-values are given for comparison with matched *EGFR*-negative controls.

Case-control analysis of subpopulations shows that the OS advantage of *EGFR*-positive patients was independent of type of *EGFR*-mutation, gender and age (Forest plot Figure 5). Comparing the different *EGFR* mutations, there was a trend in favor of exon 19 mutations (HR 0.49) compared to exon 21 mutations (HR 0.61). The few patients with exon 18 mutations had a very good survival compared to controls (HR 0.46). Similarly, there were minor trends in favor of female patients and older patients. With respect to stage, an improved OS was seen in metastasized disease, but not in localized disease. In the *EGFR* case-control population with stage I-III (n=13), 11 patients were resected in both groups. Despite adequate adjuvant therapy, the recurrence rate after resection was higher in *EGFR*-positive patients than in matched controls (7/11 [64%] vs. 5/11 [45%]). All *EGFR-*positive case-control patients initially diagnosed at stage I-III who had a recurrence received EGFR-TKI therapy. None of the comparisons between subpopulations reached statistical significance.

### EGFR-mutation: OS by TKI-treatment

**Figure 6:**
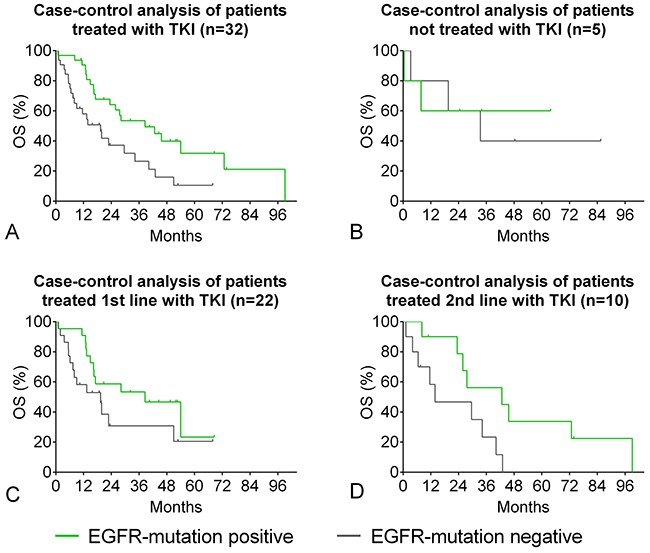
*EGFR*-positive patients: overall survival by TKI-therapy Case-control analysis of *EGFR*-positive and negative patients matched for gender, clinical stage, performance status, smoking status, and age. For patient characteristics and type of *EGFR* mutation cp. Table 3. **(A)** *EGFR*-mutation positive patients who received EGFR-TKI therapy. **(B)** *EGFR*-positive patients who did not receive EGFR-TKI therapy. **(C)** *EGFR*-positive patients who received 1^st^ line EGFR-TKI therapy. **(D)** *EGFR*-positive patients who received 2^nd^ line EGFR-TKI therapy.

A case-control analysis of EGFR-TKI treated *EGFR-*positive patients compared to negative patients is given in Figure 6. OS of *EGFR*-positive patients treated with EGFR-TKI either 1^st^ or 2^nd^ line was 38.6 months compared to 19.4 months of *EGFR*-negative controls (HR 0.47, CI 0.23-0.80, p=0.010, Figure 6A). 5-year survival was 31.9% compared to 10.9% of *EGFR*-negative controls. The survival benefit was more pronounced for patients treated with EGFR-TKI as 2^nd^-therapy (HR 0.39, CI 0.10-0.84, p=0.031, Figure 6D) than for patients treated with EGFR-TKI 1^st^ line (HR 0.56, CI 0.24-1.19, p=0.13, Figure 6C). In the few *EGFR-*positive patients not treated with EGFR-TKI, there was no difference in the OS compared to *EGFR*-negative controls (HR 0.80, CI 0.14-4.66, Figure 6B).

### *EGFR*-positive patients: resistance to TKI-treatment

**Figure 7:**
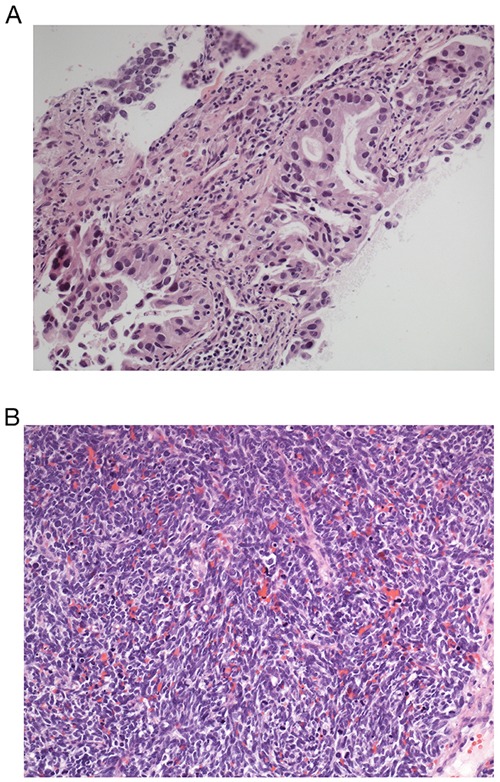
HE stains of endobronchial biopsies of patient 7 (Figure 4) **(A)** *EGFR*-positive adenocarcinoma. **(B)** Transformation into small-cell lung cancer. Magnification 400 fold.

Of eleven patients with acquired resistance to 1^st^/2^nd^ generation EGFR-TKI therapy, ten had an accessible progressive lesion and were re-biopsied. In the remaining case, liquid biopsy was used. In eight patients (73%), a T790M-resistance mutation was detected. Seven of 8 patients with del exon 19 mutation and one of three patients with exon 18 mutation had the T790M resistance mutation. In one patient, the initial re-biopsy was negative for T790M. However, a second re-biopsy taken after chemotherapy followed by erlotinib beyond progression revealed a T790M-mutant clone. In a patient with an activating *EGFR*-exon 18 mutation (c.2155G>T; p.G719C), NGS at progression on erlotinib revealed three mutations: the activating exon-18 mutation, a new T790M mutation, and a V600E mutation of the *BRAF* gene which had been undetectable by pyrosequencing in the initial biopsy. The patient responded to the third generation EGFR-TKI osimertinib and did not receive targeted therapy against the *BRAF* mutation. Another patient with extensively metastasized del exon 19-positive AC was treated 1^st^-line with erlotinib with partial remission including response of brain metastases. At progression (including progressive brain metastases and a new liver metastasis) after 7 months on erlotinib, a re-biopsy revealed a T790M resistance mutation. Whilst awaiting the result of molecular testing, the patient received one cycle of carboplatin and pemetrexed and whole-brain irradiation. After report of the T790M mutation, therapy with osimertinib was started resulting in partial remission. After four months on osimertinib, the liver metastasis was stable but primary tumor and mediastinal lymphadenopathy were rapidly progressive. Re-biopsy revealed small-cell lung cancer as the underlying cause (Figure 7). Molecular workup using NGS confirmed the continued presence of the initial *EGFR* exon 19 deletion in the SCLC biopsy with high allele frequency of 92%. However, the T790M TKI resistance mutation had become undetectable. Additionally, the tumor now carried a highly penetrant TP53 inactivating mutation (p.R248W; 92% allele frequency) which represents a common genetic variant in small-cell lung cancer [52]. One patient with adenosquamous carcinoma and a low level *EGFR* exon 18 G719C mutation (2%) responded to first-line erlotinib with stable disease for 18 months. At progression of the primary tumor, a re-biopsy revealed squamous-cell carcinoma. The *EGFR* mutation and the AC component were not detectable any more.

All patients with a T790M mutation were treated with osimertinib (n=8). There were six partial remissions and one stable disease on osimertinib, one patient had not been assessed at data bank lock. Two patients progressed after 4 and 5 months on osimertinib. After a median follow-up of 5.5 months (0.5-9.2 months), median PFS and OS have not been reached.

## DISCUSSION

The increased proportion of never-smokers and women among the *EGFR*-tested NSCLC population underlines the usefulness clinical selection criteria (i. e. predictors of the presence of a sensitizing *EGFR* mutation) leading to an enrichment of positive cases [50, 53]. Hence, the prevalence of oncogenic driver mutations in our study was higher than expected for a Caucasian patient population [33, 54, 55, 56]. For *ALK* and *BRAF*, further enrichment can be achieved by exclusion of patients tested already positive for *EGFR* and, in case of *BRAF*, for *ALK*. Our data are in line with results from the Lung Cancer Mutation Consortium [30] who found an activating EGFR mutation in 17%, an ALK translocation in 8% and a BRAF mutation in 2% in a similarly enriched predominantly Caucasian population with a high proportion of females (60%) and never-smokers (34%). The finding that about 8% of *EGFR* mutations were non-activating point mutations in exon 19 and 21 of the *EGFR* gene or activating *EFGR* mutations not conferring EGFR-TKI sensitivity underlines the necessity to check each mutation for its clinical relevance before starting EGFR-TKI treatment.

The patient selection for *EGFR*-mutation testing resulted in a population with good prognostic parameters: The stage IV AC population tested at least for *EGFR* mutation status (n=165) had a median OS of 19.8 months compared to only 9.8 months in unselected stage IV AC patients. This is not explained by differences in age (tested population: 67.2, unselected population: 67.7). However, performance status of the tested stage IV population was better than that of the unselected population (ECOG 0: 22% vs. 17%, ECOG 1: 56% vs. 52%, ECOG 2: 18% vs. 22%). Also the proportion of never-smokers or ex-smokers (72% vs. 63%) was higher among the tested population. Finally, due to enrichment, the proportion of driver-positive patients is expected to be higher in the tested population. Good performance state, non-smoker status, and presence of a driver mutation are associated with an improved prognosis and account for the improved survival of the *EGFR*-tested population.

Both in the unselected and in the case-control population, driver-positive patients had a longer OS than driver-negative patients with a HR of about 0.60. The survival advantage was confined to stage IV patients who are most likely to receive targeted therapy. An advantage in OS was seen *EGFR*-positive patients as well as in *ALK*- and *BRAF*-positive patients. This emphasizes the need to test all patients with advanced NSCLC and AC histology for drugable driver mutations. In the near future, this will be facilitated by increased availability of multiplex-testing in routine clinical practice. These techniques using next-generation sequencing test with higher sensitivity than previous methods that were based on e. g. Sanger sequencing or FISH and work particularly well with small biopsies and low tumor cellularity [46, 57, 58]. Moreover, they can also be applied for testing of liquid biopsies.

The subgroup analysis of the *EGFR* case-control population revealed an improved OS irrespective of the type of *EGFR* mutation, gender or age. In line with previous reports, *EGFR*-positive patients with exon 19 mutations had a trend to longer OS (39 months) than those with an exon 21 mutation (34 months) [5, 20]. The five patients with rare exon 18 mutations showed an even better survival, but due to small numbers, no final conclusions can be drawn [59]. Time on TKI-treatment included a median of 14 months on TKI beyond progression which prolongs disease control [60] and may, by preventing early switch to chemotherapy, positively influence patients’ quality of life [31].

In localized stages, *EGFR*-positive patients did not have an advantage in OS, although *EGFR*-positive patients received EGFR-TKI at recurrence. A potential benefit of targeted therapy may have been balanced by the higher proportion of recurrences after definitive therapy in the *EGFR*-positive group. From our data, we cannot decide whether this is a chance finding or reflects a higher risk of recurrence in driver-positive NSCLC. However, although the finding of a higher recurrence rate in *EGFR*-positive stage I-III patients is based on a small number of patients, it is tempting to speculate that cytotoxic (adjuvant or neo-adjuvant) chemotherapy might be less effective in *EGFR-*positive tumors in a curative-intent setting.

The median OS of 38.6 months found in our *EGFR*-positive population on EGFR-TKI is longer than that reported in phase IIb/III studies (19-28 months) [5, 21, 35]. Newer retrospective data show a slightly longer median OS of up to 31 months [25, 26]. TKI-treatment beyond progression which may be used more frequently in routine clinical practice than in a more rigid clinical study setting may have contributed to the increased survival [47, 48]. Moreover, in our study, the control patients (mainly stage IV NSCLC) showed a relatively long survival as well indicating good prognostic parameters in the EGFR-TKI-treated case-control population. The HR for OS of *EGFR-*positive patients compared to *EGFR*-negative controls was 0.47 if the *EGFR-*positive patients received EGFR-TKI therapy compared to 0.80 for those who did not receive EGFR-TKI-therapy. In accordance with the literature, this points to a contribution of EGFR-TKI therapy to the improved OS of *EGFR*-positive patients.

Our observation that patients receiving EGFR-TKI as 2^nd^ line therapy tend to have a longer OS than patients on 1^st^ line EGFR-TKI is in line with the French registry data but contrasts with the Chinese retrospective experience [32, 33]. However, neither study reports the clinical reason why some patients received 1^st^-line chemotherapy and other 1^st^ line chemotherapy possibly resulting in systematic imbalances between the characteristics of patients treated with EGFR-TKI 1^st^ or 2^nd^ line. This question can only be answered by a randomized clinical trial systematically comparing the treatment sequence of EGFR-TKI and chemotherapy. With current evidence of an OS advantage for patients sequentially receiving both EGFR-TKI and chemotherapy compared to either TKI or chemotherapy alone [21], it is important in clinical practice to use both EGFR-TKI and chemotherapy.

Our early experience with re-biopsy at progression on EGFR-TKI shows that re-biopsy is feasible in clinical practice and affects further treatment with detection of a treatable T790M-resistance mutation in a high proportion of cases. In the near future, increased availability of liquid biopsy and NGS will allow all EGFR-mutation positive patients who progress on EGFR-TKI to be tested for resistance mutations. Two other possible mechanisms of resistance were detected in our population: transformation into small-cell lung cancer, and a *BRAF* mutation. The clinical relevance of the *BRAF* mutation is unknown since it was detected in addition to a T790M mutation, and the patient so far did not receive targeted treatment against *BRAF*. The knowledge both on drugable activating mutations and on resistance mutations - not only in EGFR-positive NSCLC [36], but also in ALK-rearranged NSCLC [61] - is rapidly increasing. It is therefore of paramount importance for first-line and further-line therapies to perform a complete mutation screening in each eligible patient. In this context, traditional technology and sequential testing are of limited value since they require large tumor biopsies and possibly repeat biopsies in case of low tumor cellularity. The availability of new sensitive multiplex detection methods will make precision-medicine approaches available to most eligible patients and improve their quality of life and prognosis. Liquid biopsies will complement these approaches further. Moreover, the case showing transformation into SCLC strongly argues for concomitant histologic evaluation. Although some genetic events have been described recently, genetic analysis alone will most likely miss this resistance mechanism that has direct therapeutic implications. [62]

Limitations: Data are retrospective and non-randomized with a limited sample size. However, due to the large control group, exact matching of most driver mutation-positive patients was possible demonstrating significant clinical results. Only testing for *EGFR* was complete, thus the control group may include some *ALK*-, *BRAF*- or *ROS1*-positive patients. Moreover, Sanger sequencing may have missed some activating mutations which might have been found with more sensitive techniques (e. g. NGS) evolving during the study period.

Strengths: Our study provides data on diagnostic strategies, treatment patterns and OS in a current typical predominantly Caucasian lung cancer population with long-term follow-up data of up to 100 months. Since our study is a case-control analysis, we provide data from routine clinical practice on the effect of driver mutations and treatment on OS beyond anecdotal evidence of single positive cases.

In conclusion, testing for driver mutations is feasible in routine clinical practice, profoundly affects treatment and identifies patients with a good prognosis. Driver mutation-positive patients on TKI therapy in clinical routine have an OS at least as favorable as that reported in clinical studies. Our study supports current data that improved OS can be attributed to targeted therapies, and shows controlled long-term survival rates for driver-positive NSCLC that well exceed reported trial data.

## MATERIALS AND METHODS

### Patients

We recorded results of genetic testing (*EGFR*, *ALK*, *ROS1*, *BRAF*), treatment, and survival in patients diagnosed with NSCLC at our institution (lung cancer center certified by the German Cancer Society [DKG]) from January 2006 until December 2015. The databank was locked on December 31^st^, 2016. Overall survival (OS) was analyzed retrospectively in unselected patients and in case-control analyses of pairs of driver-positive and driver-negative patients individually matched for gender, smoking status, performance state, stage, and age. To avoid bias, matching was performed using only these parameters (i.e. blinded to patient number, treatment, and survival). Staging was performed according to UICC 7^th^ edition [63]. Stage matching included substage (A vs. B for stage I-III) and M-status (M1a vs. M1b for stage IV). Smoking status was self-reported. The study was approved by the local ethics committee (Landesärztekammer Baden-Württemberg F-2017-004).

### Molecular pathology

Pathological diagnosis and grading were carried out in accordance with the respective relevant WHO-guidelines [64, 65, 66]. In accordance with ESMO guidelines [67], molecular testing was performed in patients with predominant AC histology and a palliative situation (no surgical curative option, no definitive radio-chemotherapy), if TKI-therapy was considered a treatment option. Furthermore, patients with NSCLC of any histological subtype and stage were tested when our center participated in the REASON study [68] which allowed testing of patients with NSCLC irrespective of stage or histology. All molecular tests were performed by two quality controlled centers of pathology (Institute of Pathology Esslingen (certified by the national accreditation body of the Federal Republic of Germany (DAKKS), and Institute of Pathology Heidelberg, Heidelberg (accredited by the national accreditation body of the Federal Republic of Germany (DAKKS)).

*EGFR*-mutation testing has been performed since June 2009. *EGFR* testing was done from tumor biopsies with at least 20% tumor-cell content using micro-dissection of tumor cells, PCR, and Sanger sequencing of exons 18, 19, and 21 of the *EGFR* gene. Since January 2015, also exon 20 has been tested. With the availability of the third-generation EGFR-TKI osimertinib, patients with EGFR-TKI sensitive NSCLC and acquired resistance to 1^st^/2^nd^ generation EGFR-TKI therapy with an accessible progressive lesion were re-biopsied for molecular pathological workup with a focus on detection of the T790M-resistance mutation in exon 20. In one case, liquid biopsy was used for molecular pathological workup [69].

Testing for *ALK* translocation status became available in October 2012. *ALK* translocation testing was performed using FISH. On an individual patient basis, testing for *ROS1* translocation (FISH) and *BRAF* mutation (pyrosequencing) was performed. Following the concept of mutual exclusiveness of oncogenic driver mutations, tests for *ALK*, *ROS1*, and *BRAF* were only performed in AC with a negative *EGFR*-test result. Since 2016, next generation sequencing (NGS) testing both for mutations (*EGFR* incl. T790M, *BRAF*) and translocations (*ALK, ROS1*) has been used for some patients [70].

### Principles of TKI treatment in the study population

For *EGFR*-, *ALK*-, or *BRAF*-positive patients, the following principles based on experience with patients with *EGFR*-positive NSCLC were applied:

- Use of TKI beyond radiological progression until clinically relevant progression [29, 71, 72, 73].
- Dose adjustments to avoid intolerable side effects [74].
- Sequential use of different generation TKIs, e. g. 3^rd^ generation EGFR-TKI osimertinib (after demonstration of a T790M resistance mutation in a re-biopsy) following 1^st^ or 2^nd^ generation EGFR-TKIs gefitinib, erlotinib, or afatinib for *EGFR*-positive patients, or 2^nd^ generation ALK-TKI ceritinib following 1^st^ generation ALK-TKI crizotinib for *ALK* positive patients.
- Addition of local therapy, in particular radiotherapy, as clinically needed.
- Use of systemic chemotherapy in case of clinically relevant systemic progression if switch to a higher generation TKI was not possible or local therapy was insufficient to control disease.
  - If platinum-doublet chemotherapy was used, EGFR-TKI was paused following the concept of “TKI drug holiday” by Becker and colleagues [47].
  - During most of the study period, single-agent chemotherapy was combined with EGFR-TKI beyond progression to prevent disease flare described after discontinuation of EGFR-TKI [48, 75].

### Statistical methods

Kaplan-Meier plots were generated using GraphPad Prism6. Hazard ratios and significances were calculated using the log-rank test (Mantel-Cox). For age comparisons, an unpaired t-test was used (GraphPad Prism6).
